# Ambient-Dried Cellulose Nanofibril/H_2_TiO_3_ Composite Aerogel for Enhanced Lithium Capture at Seawater-Relevant pH

**DOI:** 10.3390/gels12100873

**Published:** 2026-09-28

**Authors:** Xueqiao Wang, Jing Wang, Wenxuan Wang, Ruimeng Liu, Yimin Shi, Daxin Liang

**Affiliations:** 1 School of Science, Jiamusi University, Jiamusi 154007, China; 2 Key Laboratory of Bio-Based Material Science and Technology (Ministry of Education), Northeast Forestry University, Harbin 150040, China; 18098828607@nefu.edu.cn (J.W.); wxwang@nefu.edu.cn (W.W.); liuruimeng@nefu.edu.cn (R.L.); 3 School of Materials Science and Engineering, Jiamusi University, Jiamusi 154007, China; 4 School of Chemistry and Chemical Engineering, Harbin Institute of Technology, Harbin 150001, China; 24s125126@stu.hit.edu.cn

**Keywords:** CNF, ammonium phytate, alkaline microenvironment, lithium enrichment

## Abstract

Efficient seawater lithium harvesting requires adsorbents combining high selectivity with scalable, low-energy fabrication. Although H_2_TiO_3_ lithium-ion sieves (HTO-LIS) exhibit exceptional Li^+^ selectivity, their deployment is hindered by strict alkaline requirements and reliance on energy-intensive shaping processes such as freeze-drying. Herein, we report a cellulose nanofibril (CNF) composite aerogel addressing both challenges simultaneously. Ammonium phytate (AP), a bio-derived polyphosphate, serves a dual function: as a green crosslinker for CNF and as an in situ alkalinity generator, creating a localized alkaline microenvironment around immobilized HTO nanoparticles that enables efficient topotactic H^+^/Li^+^ exchange at seawater-relevant pH 8 without requiring strongly alkaline bulk conditions. Crucially, the AP-crosslinked CNF skeleton withstands capillary forces during ambient-pressure drying, preserving a hierarchical macroporous network without freeze-drying. The resulting AP-CNF@HTO monolith achieves a Li^+^ capacity of 19.08 mg g^−1^ at pH 8, a 128.9% enhancement over pristine HTO, with excellent selectivity against competing ions in real seawater and 88.3% capacity retention after ten cycles. This work demonstrates that rational bio-based crosslinker selection can concurrently solve the alkaline microenvironment and ambient-drying challenges of HTO-LIS composites, offering an ambient-pressure-drying route with reduced reliance on energy-intensive freeze-drying for seawater lithium enrichment.

## 1. Introduction

Lithium-ion batteries dominate the fields of electrochemical energy storage and new energy transportation by virtue of their high energy density, long cycle lifespan and mature technical system. Lithium has evolved from a common industrial metal into “white petroleum”, which is vital to national energy security, industrial chain resilience and geopolitical competition. However, the rapid expansion of lithium demand is in sharp contradiction with the structural imbalance of lithium supply [1,2,3]. Current lithium resources are predominantly supplied via mineral lithium extraction and salt lake brine lithium extraction. Terrestrial lithium resources are inherently limited, so it is essential to explore diversified and sustainable alternative lithium supply strategies. As the largest lithium reservoir on Earth, seawater contains approximately 230 billion tons of lithium, which is more than 15,000 times the proven total terrestrial lithium reserves. Despite the ultra-low average lithium concentration of merely 0.17 mg L^−1^ in seawater, lithium exists as a typical ultra-trace component [2]. Nevertheless, its enormous total reserves, wide and uniform distribution, and renewable replenishment enable seawater to be recognized as the ultimate lithium resource treasury for human beings. Direct lithium extraction from seawater is an effective approach to break through terrestrial resource bottlenecks and guarantee the long-term security of the new energy industrial chain [4]. Therefore, the development of low-energy-consumption, high-efficiency, eco-friendly seawater lithium harvesting systems with engineering scale-up potential has become a crucial research direction.

Among various seawater lithium extraction technologies, the adsorption method is regarded as one of the most promising routes for industrialization due to its mild operating conditions, superior selectivity, favorable environmental compatibility, and excellent coupling performance with other processes [5,6]. Adsorbents have evolved from early organic ion-exchange resins and inorganic zeolites to lithium ion sieves (LISs) represented by manganese-based and titanium-based materials [7,8]. Notably, H_2_TiO_3_-type lithium ion sieves (HTO-LISs) possess a unique layered structure that enables lattice-level size matching and specific recognition toward Li^+^. Such structural characteristics endow HTO-LISs with a superior Li^+^/Mg^2+^ separation coefficient and excellent structural stability compared with other adsorbents, making them the core research focus of current seawater lithium extraction studies. The lithium adsorption behavior of HTO-LISs is essentially a topochemically controlled proton-lithium ion exchange reaction. Nevertheless, efficient lithium enrichment by HTO-LISs can only be achieved under sufficient OH^−^ environments. Natural seawater maintains a weakly alkaline pH range of 8.0–8.2 with an extremely limited buffer capacity, which fails to provide adequate alkalinity to sustain the high-capacity adsorption of HTO-LISs [9,10]. The addition of excessive alkaline reagents such as NaOH to adjust the pH above 10 substantially increases operational costs and poses potential secondary pollution risks. More critically, although HTO-LIS powders exhibit high specific surface areas and rapid adsorption kinetics, serious powder loss occurs during practical operation, which further elevates the overall preparation and application costs [11,12,13,14].

To address the aforementioned limitations, various polymers including polyvinyl chloride, polyvinylidene fluoride, polyacrylonitrile, and polysulfone have been adopted as carriers to immobilize HTO-LIS for lithium enrichment [15,16,17]. Nevertheless, these polymeric matrices exhibit considerable mass transfer resistance, which hinders the internal diffusion of Li^+^ toward embedded HTO-LIS and thus results in sluggish adsorption kinetics. As sustainable and widely accessible polymeric materials, biomass materials possess excellent environmental compatibility, while their inferior wet mechanical strength leads to structural collapse during cyclic operation. Previous studies have utilized chitosan as a supporting matrix for HTO-LIS immobilization [8,10,18]. However, conventional crosslinkers for aerogel fabrication usually cause environmental pollution, and pristine biomass materials suffer from insufficient chemical functionality [19]. Accordingly, current research focuses on modifying the chemical properties of biomass materials to fabricate high-performance HTO-LIS carriers [20]. For instance, carboxymethyl cellulose crosslinked with citric acid and grafted with quaternary ammonium groups has been reported to serve as a functional carrier, which reserves hydroxyl groups and reduces the optimal adsorption pH of HTO-LIS significantly [17,21]. Cellulose nanofibril (CNF), a renewable natural biomass nanomaterial, possesses a high aspect ratio, superior mechanical strength, abundant surface hydroxyl groups, and favorable biocompatibility. Three-dimensional CNF aerogel networks with ultrahigh porosity, large specific surface area, interconnected open channels and low density can be self-assembled via freeze-drying treatment. Unfortunately, pure CNF aerogels are prone to swelling and disintegration in aqueous environments. Their insufficient water stability and mechanical strength, as well as the lack of functional groups for regulating ion adsorption behavior, severely restrict their practical application in seawater lithium extraction [22,23,24]. Phosphorus-containing crosslinkers are regarded as one of the most effective strategies to construct structurally stable CNF frameworks. Unlike conventional crosslinkers that rely on freeze-drying for pore formation, ammonium phosphate has been employed to fabricate CNF aerogels with well-preserved porous structures under ambient drying conditions [25,26].

In this work, a CNF aerogel crosslinked with ammonium phytate (AP) is proposed as a multifunctional carrier for HTO-LIS immobilization that simultaneously addresses two critical bottlenecks in practical seawater lithium extraction: the energy-intensive shaping requirement and the strict alkaline condition dependency. The multiple phosphate groups in AP molecules form dense hydrogen-bonding networks and electrostatic interactions with hydroxyl groups on CNF surfaces, endowing the resulting monolithic aerogel with sufficient mechanical robustness to withstand capillary forces during ambient-pressure drying—thereby avoiding the freeze-drying step commonly employed in the fabrication of porous HTO-based composite adsorbents. Beyond its structural role, AP serves a second, equally critical function: the incompletely dissociated ammonium phosphate and ammonium hydrogen phosphate groups construct an efficient local proton buffering system within the composite. During Li^+^ uptake, HTO-LIS releases protons via the topotactic H^+^/Li^+^ exchange reaction; the adjacent AP phosphate groups instantly capture and neutralize the generated H^+^, preventing abrupt local pH reduction and thereby maintaining a stable weakly alkaline microenvironment inside the adsorbent. This in situ proton buffering enables efficient ion exchange directly at seawater-relevant pH (pH = 8) without requiring strongly alkaline bulk conditions—a strategy not previously reported for HTO-LIS composites. Experimental results demonstrate that the saturated Li^+^ adsorption capacity of the AP-CNF@HTO composite aerogel is substantially improved compared with pristine HTO-LIS powders under simulated weakly alkaline seawater conditions (pH = 8). Moreover, the composite retains efficient Li^+^ capture capability in simulated seawater containing competing ions (Na^+^, K^+^, Mg^2+^, Ca^2+^). After ten complete adsorption–acid desorption cycles, the composite retains 88.3% of its initial adsorption capacity, with no obvious structural collapse or excessive active-component loss.

## 2. Results and Discussion

Figure 1 demonstrates the fabrication procedure and lithium extraction mechanism of the AP-CNF@HTO composite. Using TEMPO-oxidized CNF as the matrix and ammonium phytate (AP) as both the crosslinking agent and pH-buffering functional medium, porous aerogels are readily prepared through ethanol solvent replacement and ambient-temperature drying. This strategy effectively avoids the high-cost drying processes adopted in conventional aerogel fabrication. AP is stably crosslinked with CNF via electrostatic interactions and P–O–C covalent bonds, enabling the uniform anchoring of phosphate buffering groups on the three-dimensional fibrous network, while HTO nanoparticles are immobilized by physical encapsulation. The titania crystal structure of HTO realizes specific lithium recognition through topological size sieving. The phosphate groups of AP can in situ neutralize protons released during the ion exchange reaction, constructing a favorable localized alkaline microenvironment under the weakly alkaline background of natural seawater. Synergized with the mass transfer and lithium pre-enrichment effects of the porous carrier, the composite material achieves high-capacity, high-selectivity, and long-cycle-stable lithium extraction from seawater.

Figure 2 presents the FT-IR spectra of pure CNF, AP-CNF, and AP-CNF@HTO composites. The broad absorption band located at 3200–3600 cm^−1^ corresponds to the stretching vibration of hydroxyl and amino groups. Compared with pure CNF, the AP-CNF sample exhibits significantly reduced absorption intensity and flattened peak shape, which is attributed to the formation of abundant hydrogen bonds between phosphate hydroxyl groups/NH_4_^+^ on AP molecules and hydroxyl [27] groups of CNF. The consumption of free hydroxyl groups restricts the free stretching of hydroxyl bonds, which intuitively verifies the intermolecular crosslinking reaction between AP and CNF. The further weakened absorption intensity of AP-CNF@HTO is caused by the incorporation of numerous Ti–O bonds on HTO-LIS into the hydrogen bond network, which strengthens the overall intermolecular interactions of the aerogel. New absorption peaks emerging at 1600–1650 cm^−1^ for AP-CNF and AP-CNF@HTO are assigned to the N–H bending vibration of AP, confirming the successful crosslinking of CNF by AP and the stabilized integral structure of the aerogel. Multiple split characteristic peaks newly observed in the range of 1050–1200 cm^−1^ for AP-CNF originate from the stretching vibration of P–O, P=O, and P–O–C bonds in phytate molecules, directly demonstrating the successful grafting of AP onto the three-dimensional CNF network. Notably, AP-CNF@HTO displays an intense and broad absorption valley near 1000 cm^−1^, which is ascribed to the stretching vibration of Ti–O and Ti–O–Ti metal-oxygen bonds inside HTO-LIS. Superposition of these characteristic signals masks the original characteristic peaks of AP. These spectral features support the successful incorporation of HTO into the AP-CNF composite. The spatial distribution of HTO within the aerogel network is further evaluated by SEM and EDS mapping [28,29].

Figure 3 displays the SEM and EDS-mapping results of the AP-CNF aerogel. After crosslinking with ammonium phytate, TEMPO-oxidized CNF nanofibers intertwine and overlap with each other to construct a continuous and interconnected lamellar three-dimensional porous architecture. Numerous mesopores and macropores with varying sizes are uniformly distributed on the material surface, demonstrating the feasibility of the facile fabrication strategy combining liquid nitrogen rapid freezing shaping, impregnation crosslinking, and natural drying. Structurally stable aerogels can be successfully prepared without high-cost and time-consuming freeze-drying procedures. EDS-mapping results reveal the homogeneous co-distribution of N and P elements across the entire aerogel region without element aggregation or local deficiency. This phenomenon intuitively confirms that ammonium phytate is uniformly grafted onto the three-dimensional CNF skeleton via intermolecular interactions rather than simple physical surface adsorption. Such structural characteristics provide a reliable structural foundation for the in situ construction of an alkaline buffering microenvironment within the material and the reduction of external alkali dosage during lithium extraction.

Figure 4 shows the SEM and EDS-mapping images of the AP-CNF@HTO composite. The three-dimensional interconnected porous skeleton of AP-CNF is well preserved after HTO loading. High-magnification SEM observations reveal that HTO nanoparticles are uniformly dispersed on the surface of cellulose lamellas without obvious agglomeration. The interconnected porous channels guarantee efficient mass transfer of metal ions in aqueous solutions, while the crosslinked network physically confines HTO nanoparticles, endowing the composite material with excellent cycling stability. EDS-mapping results intuitively verify that the HTO active adsorption components are uniformly incorporated into the aerogel network, confirming the successful fabrication of integrated composite adsorbents. A high spatial overlap between phosphate buffering groups (P) and lithium adsorption active sites (Ti) is observed within the material. This structural feature enables the in situ construction of an internal alkaline buffering microenvironment, which activates the high lithium adsorption performance of HTO under the weakly alkaline conditions of natural seawater and substantially reduces alkali consumption and secondary pollution risks during seawater lithium extraction.

Figure 5a displays the XRD patterns of pure HTO, AP-CNF, and AP-CNF@HTO composites. The diffraction peaks of AP-CNF observed at 2θ of 10–25° correspond to the characteristic peaks of cellulose, with no sharp crystalline phase signals detected. By contrast, pure HTO presents distinct and sharp diffraction peaks, demonstrating the successful synthesis of HTO nanoparticles. For the AP-CNF@HTO composite, the crystalline structure of HTO is well maintained without obvious diffraction peak shifts, which confirms that the mild composite fabrication process does not damage the lithium-exchange crystal skeleton of HTO. Combined with the AFM results in Figure S1, the stable crystalline structure and uniform dispersion of HTO on the CNF skeleton are further verified. Figure S2 shows the X-ray photoelectron spectroscopy (XPS) spectra of AP-CNF@HTO, and Figure 5b–f correspond to the high-resolution deconvoluted spectra of C 1s, N 1s, O 1s, P 2p and Ti 2p, respectively. The C 1s spectrum verifies that the cellulose nanofiber skeleton is well preserved, and ammonium phytate is chemically crosslinked with cellulose nanofibers via phosphorus–oxygen–carbon phosphate ester bonds. The N 1s spectrum confirms that ammonium phytate is stably anchored on the fiber network through electrostatic interactions. Abundant phosphate ions and phosphohydroxy phosphate buffering functional groups are detected in the O 1s and P 2p spectra, which confirm the presence of phosphate-containing buffering functional groups in the AP-CNF@HTO composite [30,31]. Furthermore, the characteristic spin–orbit doublet assigned to Ti^4+^ is visible in the Ti 2p spectra, demonstrating that the titanium-oxygen framework of HTO remains intact throughout composite fabrication. The Ti 2p characteristic peaks exhibit a shift to lower binding energy of ~0.2–0.4 eV, verifying the replacement of hydrogen ions with lithium ions in the sieve structure. After adsorption, a Li 1s signal is detected in the material (Figure S3), confirming Li^+^ uptake by AP-CNF@HTO [18,32].

Figure 6 illustrates the zeta potential test results of CNF and AP-CNF. TEMPO-oxidized CNF exhibits strongly negative surface charges ranging from −40 mV to −50 mV due to abundant surface carboxyl groups, which endow the CNF colloid with excessively high stability, thereby resulting in poor moldability and aqueous structural instability. After crosslinking with ammonium phytate, the negative surface charges of AP-CNF are neutralized by ammonium ions, and the zeta potential shifts significantly toward zero. This variation directly verifies the stable electrostatic crosslinking reaction between ammonium phytate and CNF. The charge neutralization effect promotes the self-assembly of cellulose nanofibers into a three-dimensional porous aerogel architecture and firmly immobilizes phosphate buffering groups within the carrier network, providing an electrochemical foundation for the in situ construction of an internal alkaline buffering microenvironment. Furthermore, the weakly negatively charged carrier can electrostatically enrich lithium ions and uniformly disperse HTO active nanoparticles, synergistically improving the lithium adsorption performance of the composite in seawater systems.

Figure 7a presents the specific surface area characteristics of pure HTO, AP-CNF, and AP-CNF@HTO samples. Pure HTO powder possesses the highest specific surface area and most developed pore structure, yet it suffers from severe particle agglomeration and material loss due to the absence of a rigid supporting skeleton. Ammonium phytate-crosslinked AP-CNF aerogels are defined as low-specific-surface-area organic porous carriers, which primarily function as structural skeletons and platforms for immobilizing buffering functional groups. By integrating the advantages of the two components, the AP-CNF@HTO composite achieves a significantly improved specific surface area of 29.376 m^2^ g^−1^ compared with the pristine AP-CNF carrier. The adsorption isotherms confirm the successful fabrication of interconnected hierarchical macro-mesopore structures within the composite. Such a unique porous architecture effectively disperses HTO nanoparticles, eliminates particle agglomeration, and reduces the mass transfer resistance of lithium ions in seawater. Meanwhile, phosphate buffering groups and lithium exchange active sites are uniformly distributed throughout the pore channels, synergistically enhancing the lithium adsorption capacity and cycling stability under weakly alkaline seawater conditions. This provides critical structural support for the facile fabrication of composite aerogels toward practical seawater lithium extraction.

Figure 7b displays the thermal stability of the three samples characterized by thermogravimetric analysis. Pure CNF, as a pristine organic biomass material, exhibits low bound water content, a higher thermal decomposition temperature, and negligible inorganic residues at high temperatures. After crosslinking with ammonium phytate, the resultant AP-CNF contains abundant hydrophilic groups, which advance the thermal degradation temperature and increase the residual phosphorus oxide content at high temperatures, verifying the formation of a stable crosslinked network between AP and CNF. Upon the loading of titanium-based HTO lithium-ion sieves, the AP-CNF@HTO composite delivers a prominently elevated residual mass across the entire temperature range with the highest high-temperature residue, which confirms the successful incorporation of thermally stable inorganic HTO components. All three samples only undergo the desorption of adsorbed water below 200 °C without thermal decomposition of organic skeletons or functional groups. These TGA results characterize the thermal decomposition behavior and thermal sta-bility of the prepared materials [33,34,35].

Figure 8 illustrates the Li^+^ adsorption performances of pure HTO and AP-CNF@HTO under varying pH conditions (Equation (S1)). Experimental results confirm that the AP-CNF carrier constructs an in situ alkaline buffering microenvironment through the phosphate groups of ammonium phytate, which remarkably improves the lithium adsorption capacity of HTO under the weakly alkaline condition of natural seawater (pH ≈ 8). Different from pure HTO, the composite material avoids the addition of excessive strong alkali, thereby reducing the operational cost and environmental pollution risks of the seawater lithium extraction process. Under high-pH strongly alkaline conditions, pure HTO and AP-CNF@HTO exhibit consistent Li^+^ adsorption performance. This indicates that the pH buffering effect is the core advantage of AP-CNF@HTO in weak alkaline systems. Therefore, all subsequent experiments are performed at pH = 8.

The variation of lithium adsorption capacity with adsorption time is presented in Figure 9a. Pure AP-CNF carrier exhibits negligible lithium adsorption capability. The three-dimensional porous network of AP-CNF enables uniform dispersion of hydrogen titanium oxide (HTO) particles and constructs interconnected mass transport channels. Meanwhile, the carrier surface carries a weak negative potential, which can enrich lithium ions via electrostatic interactions, greatly accelerating the adsorption kinetic rate and raising the saturated adsorption capacity to 19.08 mg g^−1^. FTIR characterization of AP-CNF@HTO after adsorption revealed characteristic absorption peaks associated with Li-O bonds, which corroborates the proposed adsorption mechanism. Meanwhile, the -OH absorption peaks of AP-CNF@HTO intensified after adsorption, indicating the formation of an internal alkaline microenvironment (Supplementary Figure S4). This strategy effectively addresses the industrial bottlenecks of pure HTO powder, including severe particle agglomeration, low mass transfer efficiency, and limited adsorption capacity, thereby fully demonstrating the critical functional role of the AP-CNF composite carrier. To further elucidate the lithium adsorption mechanism of the prepared materials, adsorption kinetic fitting was conducted based on Formulas (1) and (2). Figure 9b,c display the fitting results of the pseudo-first-order kinetic model and the linear fitting curves of the pseudo-second-order kinetic model, respectively, with the detailed fitting parameters summarized in Table S1. The kinetic fitting results show that the lithium adsorption processes of both pure HTO and AP-CNF@HTO are well described by the pseudo-second-order kinetic model, which is consistent with the intrinsic topological H^+^/Li^+^ ion-exchange behavior of HTO crystals and is highly consistent with the intrinsic adsorption mechanism of titanium-based lithium ion sieves. Compared with pristine HTO powder, the superior adsorption performance of AP-CNF@HTO is attributed to multiple synergistic effects. The interconnected hierarchical pore structure reduces the diffusion resistance of lithium ions and increases the diffusion rate constant (k). Meanwhile, the in situ neutralization of H^+^ generated during ion exchange by phosphate buffering groups maintains a stable local alkaline microenvironment, which enhances the equilibrium adsorption capacity by more than two times. Fitting was carried out using the Weber–Morris intra-particle diffusion model (Equation (S2)), and the results are displayed in Figure S5. The adsorption process of Li^+^ on AP-CNF@HTO and HTO can be divided into two stages. The first stage is liquid film diffusion, during which Li^+^ quickly passes through the boundary layer at the solid–liquid interface, resulting in a rapid increase in adsorption capacity. The second stage is the intra-particle diffusion stage: Li^+^ diffuses into the pore channels of the adsorbent, the adsorption rate slows down and gradually approaches equilibrium, revealing that liquid film diffusion also affects the adsorption process. AP-CNF possesses negligible lithium adsorption capacity, and the lithium adsorption activity of the composite comes from the HTO component. AP-CNF@HTO presents a larger slope, suggesting that composite modification effectively accelerates the mass transfer rate of Li^+^ and enhances adsorption performance. The extremely high linear correlation coefficient of 0.9992 for the pseudo-second-order kinetic model indicates good agreement between the experimental kinetic data and the fitted pseu-do-second-order model. The experimental data were further fitted using the Elovich model (Equation (S3)), and the fitting results are presented in Figure S6. The Elovich fitting suggests heterogeneous adsorption behavior at the solid–liquid interface. Compared with pure HTO, AP-CNF@HTO exhibits a faster initial Li^+^ adsorption rate, indicating enhanced Li^+^ adsorption kinetics after composite modification. This composite material enables efficient and sustained lithium ions exchange in weakly alkaline seawater environments, which indicates that chemical ion exchange is correlated with the adsorption capacity and kinetic rate of the material [26,36,37].

(1) $$\ln\left(q_{e}-q_{t}\right)=\ln q_{e}-k_{1}t$$

(2) $$\frac{t}{q_{t}}=\frac{1}{k_{2}q_{e}^{2}}+\frac{t}{q_{e}}$$

Figure 9d presents the variation in equilibrium adsorption capacity of AP-CNF@HTO with initial lithium concentration at different temperatures. The adsorption capacity follows the order of 45 °C > 35 °C > 25 °C at identical initial concentrations. The isothermal adsorption data were further fitted via Langmuir and Freundlich models based on Formulas (3) and (4) (Figure 9e,f). The satisfactory fitting degree of the Langmuir model suggests that lithium adsorption is predominantly associated with the specific topological active sites of HTO crystals. The AP-CNF aerogel merely functions for nanoparticle dispersion, pH buffering, and mass transfer promotion, without inducing non-specific multilayer physical adsorption, thereby avoiding the undesired adsorption of competing metal ions in complex aqueous systems. Severe micropore blockage caused by the agglomeration of pure HTO powder leads to a much lower practical saturated adsorption capacity than the theoretical Langmuir value. In contrast, the hierarchical interconnected pore structure of AP-CNF uniformly disperses HTO nanoparticles and constructs a favorable alkaline microenvironment, enabling the practical adsorption capacity of the composite to approach the theoretical limit of the model. This constitutes the quantitative isothermal evidence for the superior adsorption performance of the composite aerogel over pure HTO powder. Furthermore, the Freundlich model parameter n is greater than 2, revealing the excellent adsorption performance of the material for low-concentration lithium ions, which is highly suitable for lithium enrichment in natural low-concentration seawater. The equilibrium adsorption isotherm data were nonlinearly fitted by the Redlich-Peterson model (Equation (S6)), shown in Figure S7. The fitted parameters were A = 2.3433, B = 0.1372, and η = 0.9165. The η value between 0 and 1 confirms the physical significance of the model. A high coefficient of determination (R^2^ = 0.9947) and good agreement between experimental data points and the model-fitted curve demonstrate that the Redlich-Peterson model well describes the isothermal adsorption behavior of the studied system. The η value close to unity suggests that the adsorption was dominated by Langmuir-type homogeneous monolayer adsorption, accompanied by slight heterogeneity of adsorption sites on the adsorbent surface, reflecting the combined characteristics of Langmuir and Freundlich isotherms [9,38,39].

(3) $$\frac{C_{e}}{q_{e}}=\frac{C_{e}}{q_{m}}+\frac{1}{q_{m}\times K_{L}}$$

(4) $$\ln q_{e}=\ln K_{F}+\frac{1}{n}\ln C_{e}$$

Figure 10a shows the adsorption capacity of AP-CNF@HTO toward different metal ions in a multi-ion mixed solution at pH = 8. Experimental results indicate that both pure HTO and AP-CNF@HTO exhibit prominent specific adsorption capability for Li^+^, while negligible adsorption is observed for other coexisting competitive ions. This demonstrates that the topological size sieving effect of HTO crystals serves as the core origin of the excellent lithium selectivity of the adsorbent. After modification with ammonium phytate-crosslinked carriers, no obvious non-specific adsorption of impurity ions occurs on AP-CNF@HTO, confirming that the mild composite fabrication process completely preserves the intrinsic ion-selective crystal structure of HTO. Notably, the in situ phosphate buffering microenvironment constructed by the AP-CNF carrier eliminates the inhibitory effect of protons generated during ion exchange, endowing the composite aerogel with superior lithium extraction performance compared with pure HTO powder. Figure 10b presents the extraction efficiency of various cations by AP-CNF@HTO in real natural seawater collected from the Bohai Sea. The composite aerogel achieves a lithium extraction efficiency of over 99% for ultra-trace Li^+^ in natural seawater. In contrast, the extraction efficiencies of coexisting ions (Na^+^, Mg^2+^, Ca^2+^, K^+^, and Sr^2+^), whose concentrations are thousands to 50,000 times higher than that of lithium, are all lower than 5%. Particularly, nearly no co-adsorption of Na^+^ and Mg^2+^ is detected. The in situ alkaline phosphate buffering microenvironment of AP-CNF@HTO can neutralize protons released during adsorption and eliminate the adsorption inhibition effect under the weakly alkaline conditions of natural seawater. Combined with the electrostatic pre-enrichment effect of the three-dimensional porous network toward lithium ions, nearly complete capture of low-concentration lithium is successfully realized.

Figure 11 presents the saturated adsorption capacity of AP-CNF@HTO during ten cyclic adsorption tests after HCl elution, together with the Ti dissolution loss rate of HTO active components. The AP-CNF@HTO composite retains 88.3% of its initial lithium adsorption capacity after ten cycles with only mild and gradual performance attenuation. The dissolution rate of titanium derived from HTO active sites remains consistently lower than 0.7% throughout the whole cycling process, which demonstrates the outstanding acid corrosion resistance of the HTO crystalline structure. These results confirm that the integrated composite aerogel fabricated in this work effectively overcomes the industrial limitations of pure HTO powder, including difficult recovery and short cycle lifespan. The composite can sustain stable lithium enrichment performance during the integrated seawater adsorption and acid regeneration process, endowing it with promising application prospects for large-scale seawater lithium extraction.

**Figure 11:**
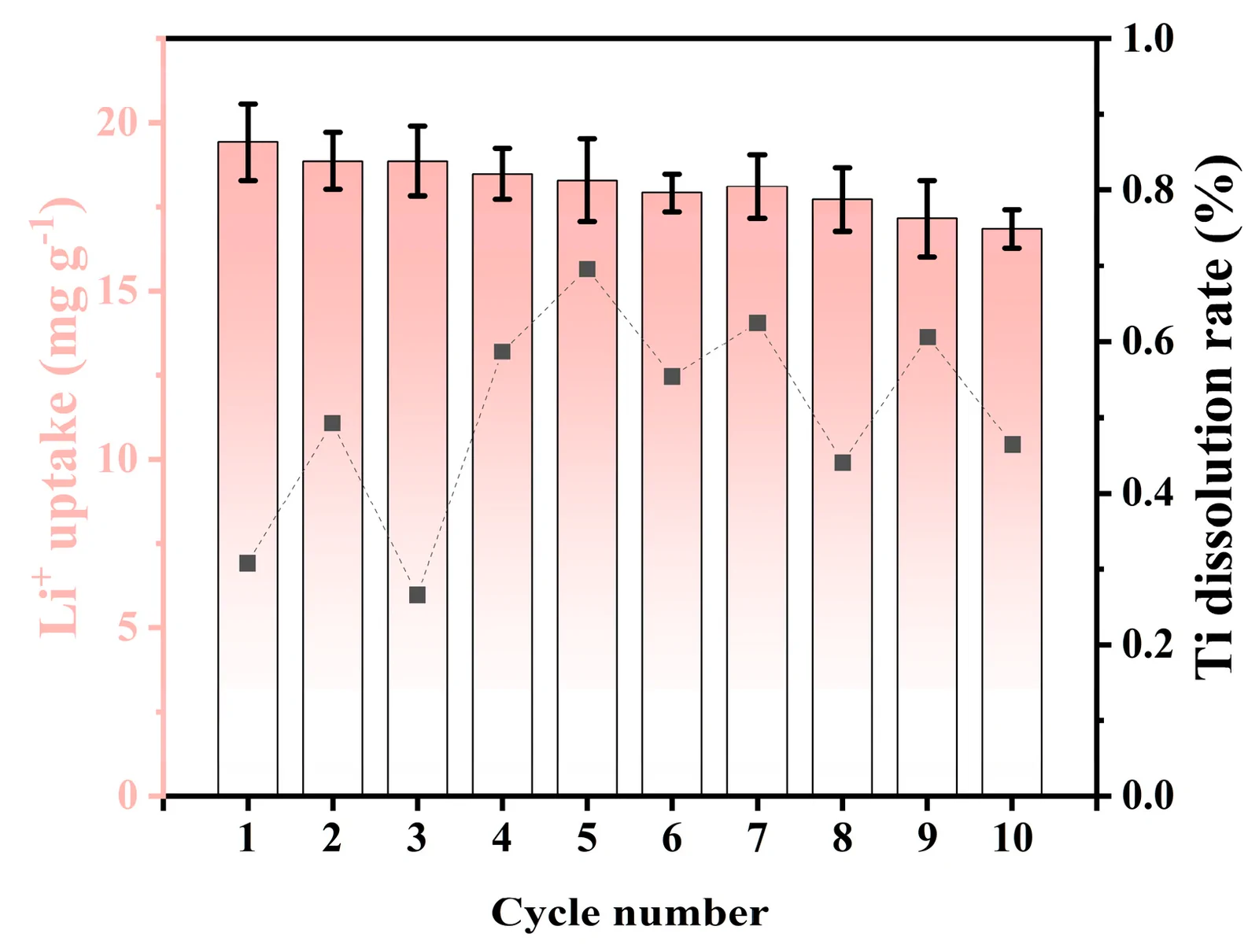
Adsorption capacity of AP-CNF@HTO during ten cyclic adsorption and the Ti dissolution loss rate of HTO active components.

## 3. Conclusions

In this study, AP-CNF@HTO composite aerogel was successfully fabricated through a simple forming process combined with ambient-temperature drying. This method effectively removes the requirement for freeze-drying technology adopted in conventional aerogel fabrication. As a multifunctional crosslinker, ammonium phytate constructs stable hydrogen bonding and covalent crosslinking networks to reinforce the structural stability of CNF aerogels, while introducing abundant phosphate buffering groups to establish an in situ alkaline microenvironment inside the composite. Under the natural weakly alkaline seawater condition (pH = 8), this unique structural design can effectively neutralize hydrogen protons released during the HTO-based lithium ion exchange reaction, achieving a prominent lithium adsorption capacity of 19.08 mg g^−1^. Benefiting from the hierarchical macro-mesoporous structure of the CNF skeleton, HTO nanoparticles are uniformly dispersed without agglomeration, reducing the mass transfer resistance of lithium ions and greatly improving the adsorption kinetics and capacity. The AP-CNF@HTO composite realizes an ultra-trace lithium capture efficiency of over 99% in real seawater and retains excellent adsorption performance after repeated adsorption–desorption cycles. The AP-CNF@HTO adsorbent provides a novel and reliable technical pathway for the efficient and sustainable enrichment of lithium resources from seawater.

## 4. Materials and Methods

### 4.1. Materials

TEMPO-oxidized cellulose nanofibril suspension (CNF, carboxyl content of 1.46 mmol g^−1^) was purchased from Tianjin Mujo Biotechnology Co., Ltd. (Tianjin, China). Phytic acid (50% aqueous solution) was obtained from Shanghai Aladdin Biochemical Technology Co., Ltd. (Shanghai, China). Titanium dioxide (anatase, ≥99.8%), anhydrous lithium acetate (CH_3_COOLi, 99%), lithium chloride (LiCl, ≥99%), potassium chloride (KCl, ≥99.8%), sodium chloride (NaCl, ≥99.8%), anhydrous calcium chloride (CaCl_2_, ≥96%), magnesium chloride hexahydrate (MgCl_2_·6H_2_O, ≥99.7%), and ammonia solution (NH_3_·H_2_O, mass fraction of 25.0–28.0%) were all supplied by Shanghai Aladdin Biochemical Technology Co., Ltd. Hydrochloric acid (HCl, mass fraction of 36.0–38.0%) was purchased from Sinopharm Chemical Reagent Co., Ltd. (Shanghai, China). All reagents were used directly without further purification throughout the experiments. The characterization was shown in Supplementary Materials.

### 4.2. Materials Preparation

Synthesis of HTO Lithium ions Sieve. Lithium titanate (LTO) precursor was first synthesized via a facile ball-milling and high-temperature calcination route. Anatase TiO_2_ and anhydrous lithium acetate were thoroughly ground in a ball mill at a Ti/Li molar ratio of 1:2 to achieve uniform mixing. The homogeneous powder mixture was calcined in a muffle furnace at 700 °C for 3 h with a heating rate of 5 °C min^−1^ to obtain LTO powder. Subsequently, 2 g of the as-prepared LTO powder was dispersed in 1000 mL of 0.2 mol L^−1^ HCl solution and continuously stirred for 24 h to complete the proton exchange reaction. The resultant product was filtered and rinsed with deionized water five times until the filtrate reached a neutral pH. Finally, the solid residue was dried in an oven at 60 °C to obtain the hydrogen titanium oxide (HTO).

Synthesis of Ammonium Phytate (AP). Ammonium phytate was prepared through a typical synthetic route. Briefly, a 50 wt% phytic acid aqueous solution and urea were added into a three-necked flask at a molar ratio of 1:6. The mixture was stirred at 110 °C for 1 h, followed by heating to 120 °C and maintaining for 15 min. After cooling to room temperature, ammonia solution was slowly dropped into the reaction system under continuous stirring until the pH value stabilized at 5.0, yielding a homogeneous AP solution.

Fabrication of AP-CNF and AP-CNF@HTO Aerogels. First, 50 mL of 1 wt% TEMPO-oxidized CNF suspension with uniform dispersion was prepared. A total of 0.5 g of HTO powder was added to the CNF suspension under ultrasonic treatment, and ultrasonic dispersion was continued for 20 min to achieve homogeneous HTO distribution. The well-mixed suspension was rapidly frozen and shaped using liquid nitrogen. The obtained solid gel was vacuum-impregnated in 2 wt% AP solution for 1 h to realize crosslinking modification. After impregnation, solvent replacement was performed in ethanol, and the treated gel was naturally dried for 6 h to obtain the AP-crosslinked CNF@HTO composite aerogel (AP-CNF@HTO). For comparison, pure AP-crosslinked CNF aerogel (AP-CNF) was fabricated through the identical procedure without the addition of HTO powder.

### 4.3. Adsorption Experimental Tests

Pretreatment of Adsorbent. Prior to adsorption experiments, the prepared aerogel adsorbents were pretreated with 0.05 M NaOH solution. The aerogels were soaked in alkaline solution for 1 h and then rinsed repeatedly with deionized water until a neutral surface pH was achieved. This pretreatment was used to condition the adsorbent surface prior to subsequent lithium adsorption.

Adsorption Kinetics and Batch Adsorption Experiments. Lithium chloride was adopted as the lithium source to investigate the lithium adsorption behavior of AP-CNF@HTO. Typically, 100 mg of the adsorbent was dispersed in lithium chloride solution with an initial Li^+^ concentration of 100 mg L^−1^. The adsorption reaction was carried out in a water-bath shaking oven at 25 °C. During the adsorption process, 5 mL of the suspension was withdrawn at preset time intervals. The collected solution was diluted and analyzed by ICP-MS to determine the residual Li^+^ concentration. The adsorption capacity was calculated according to the established Formula (1). To explore the pH-dependent adsorption performance, the solution pH was precisely adjusted using dilute HCl and NH_4_OH solutions, and the adsorption capacities at different pH values were systematically compared. For kinetic investigation, lithium solutions with varying initial concentrations were applied, and the time-dependent lithium concentration evolution was continuously monitored until adsorption equilibrium. The experimental data were fitted with pseudo-first-order and pseudo-second-order Weber–Morris and Elovich kinetic models to analyze the adsorption kinetics and mass-transfer behavior.

Adsorption Isotherm Measurements. Adsorption isotherm experiments were conducted under consistent conditions with kinetic tests. The adsorption performance of AP-CNF@HTO at different equilibrium lithium concentrations was measured to explore the saturated adsorption behavior and adsorption thermodynamics.

Ion Selectivity Test. To evaluate the anti-interference capability and lithium selectivity of the composite adsorbent, a multi-ion mixed solution containing Li^+^, K^+^, Na^+^, Ca^2+^, and Mg^2+^ was prepared. The distribution coefficient (K) of each metal ion and the separation factor (α) between lithium and competing ions were calculated to quantitatively assess the selective lithium extraction performance.

Desorption and Regeneration Experiments. After lithium saturation adsorption, the spent adsorbent was immersed in 0.1 M HCl solution for 12 h to accomplish sufficient desorption. The regenerated aerogel was rinsed with deionized water and dried for subsequent cyclic adsorption–desorption tests.

Real Seawater Adsorption Experiment. Natural seawater was sampled from the Bohai Sea for practical lithium extraction evaluation. The initial lithium concentration in raw seawater was determined to be 0.209 mg L^−1^ via ICP-MS, and the original seawater pH was measured as 8.25 using a pH meter. The lithium enrichment performance of AP-CNF@HTO in real seawater was tested under ambient conditions to verify its practical application potential.

## Figures and Tables

**Figure 1:**
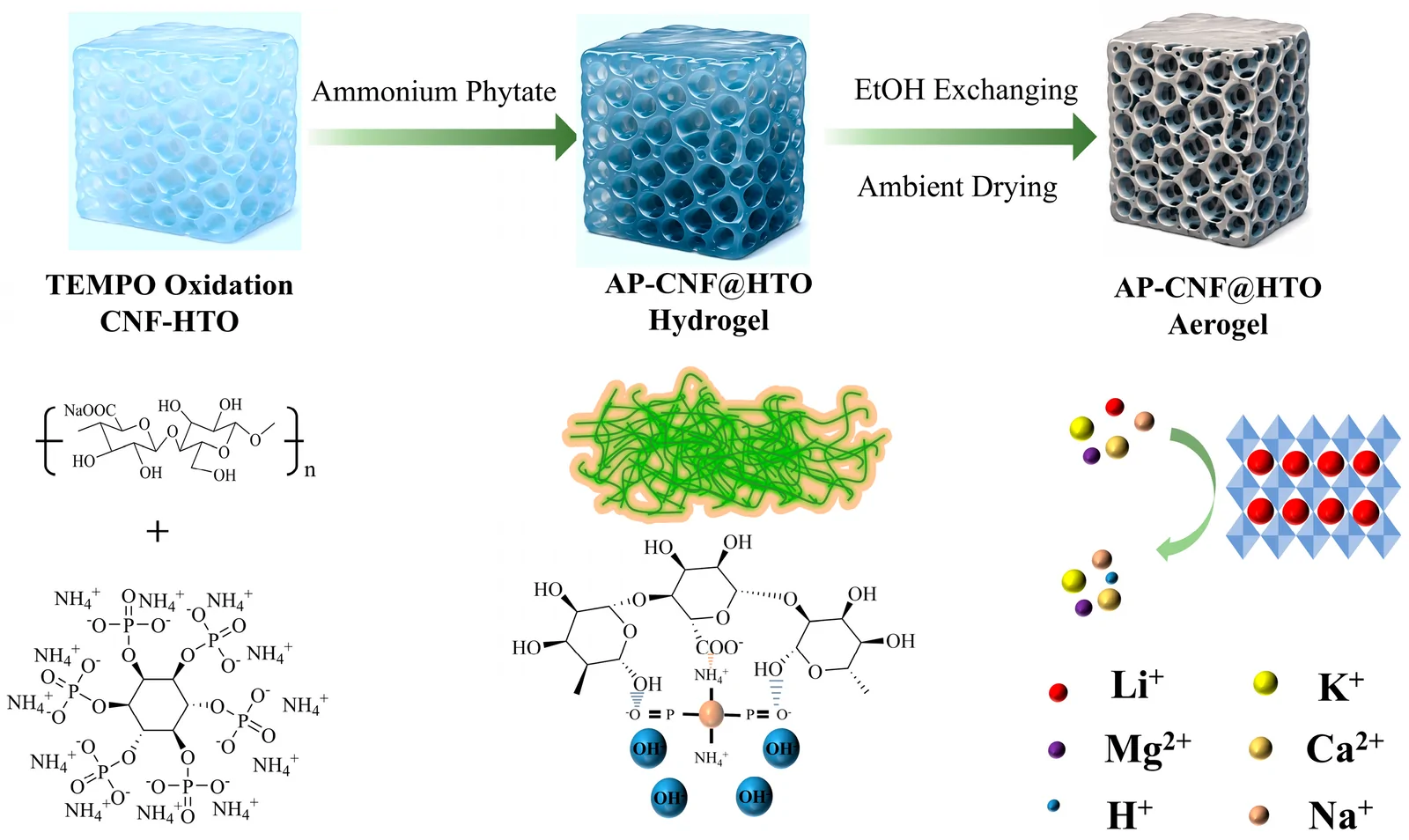
Preparation process and lithium extraction mechanism of AP-CNF@HTO.

**Figure 2:**
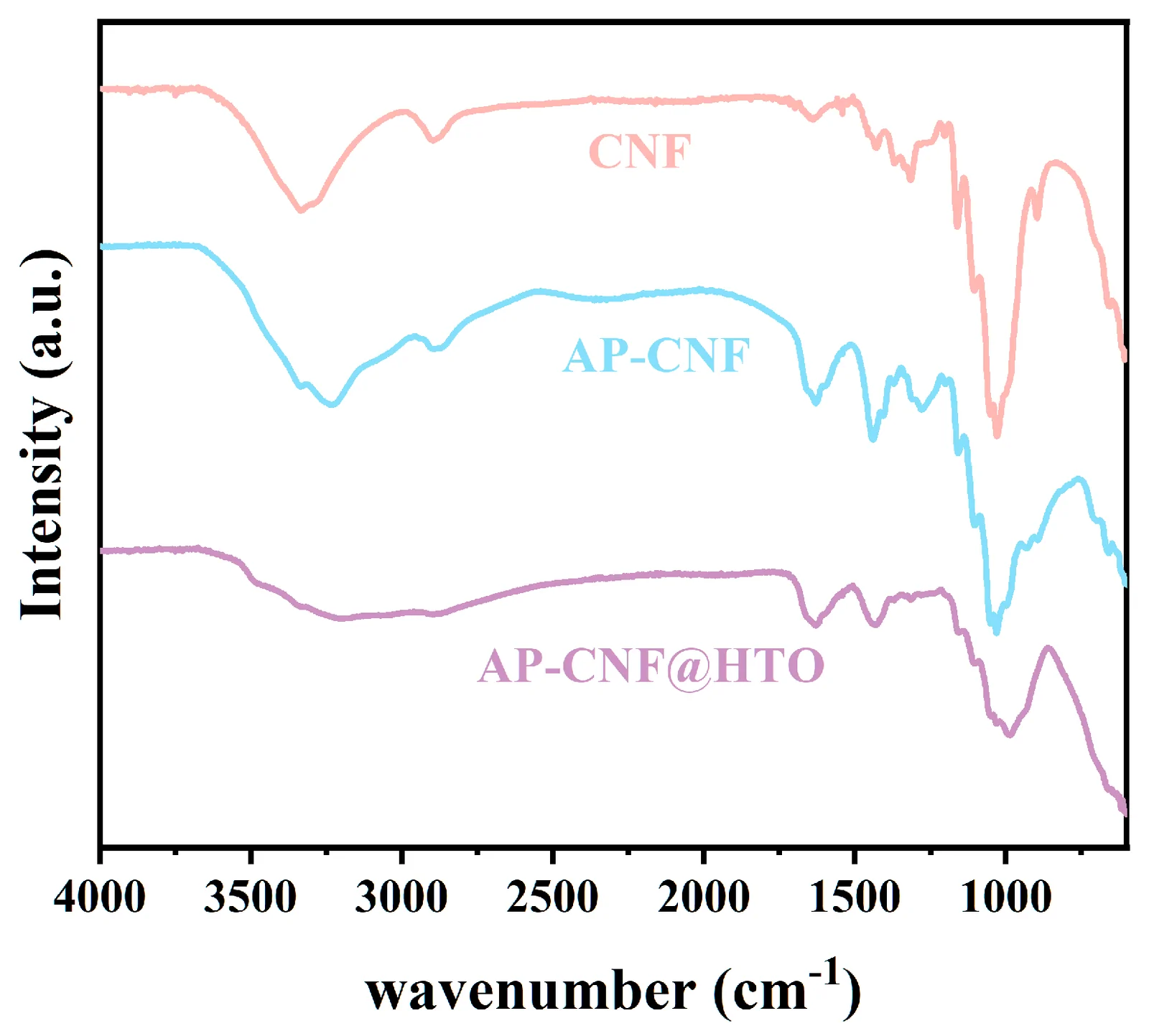
FT-IR spectra of pure CNF, AP-CNF, and AP-CNF@HTO composites.

**Figure 3:**
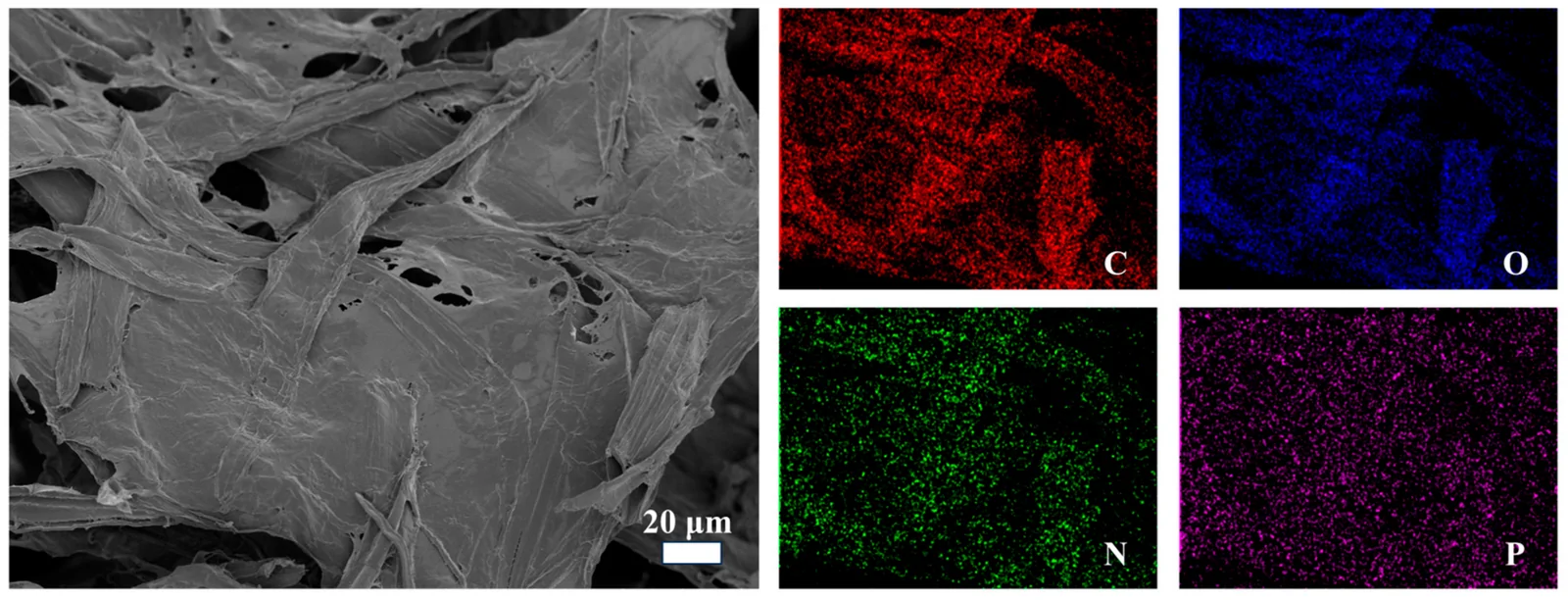
SEM and EDS mapping of the AP-CNF aerogel.

**Figure 4:**
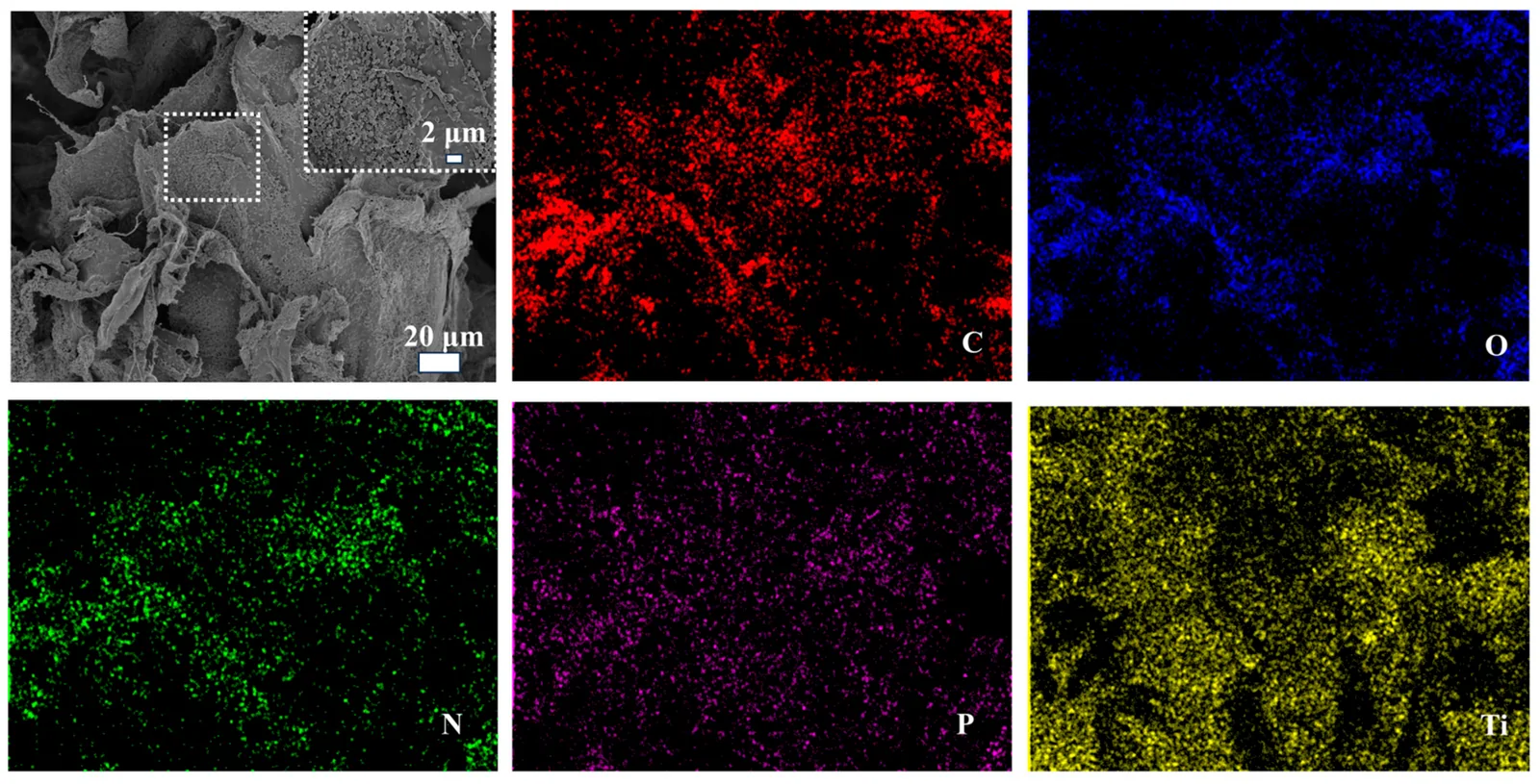
SEM and EDS-mapping of the AP-CNF@HTO.

**Figure 5:**
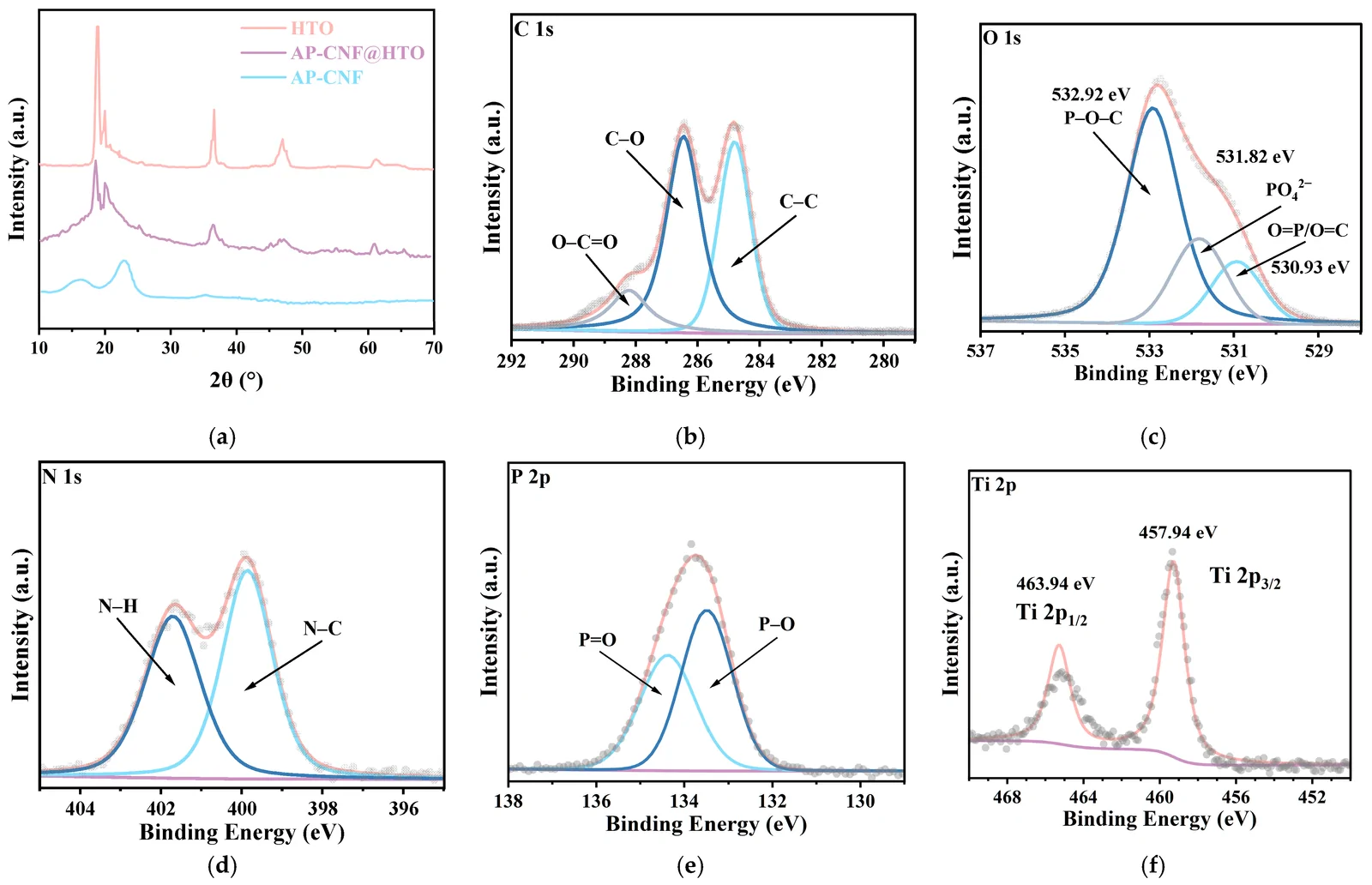
(**a**) XRD patterns of pure HTO, AP-CNF, and AP-CNF@HTO. High-resolution deconvoluted XPS spectra of AP-CNF@HTO, (**b**) C1s, (**c**) O1s, (**d**) N1s, (**e**) P2p, and (**f**) Ti2p.

**Figure 6:**
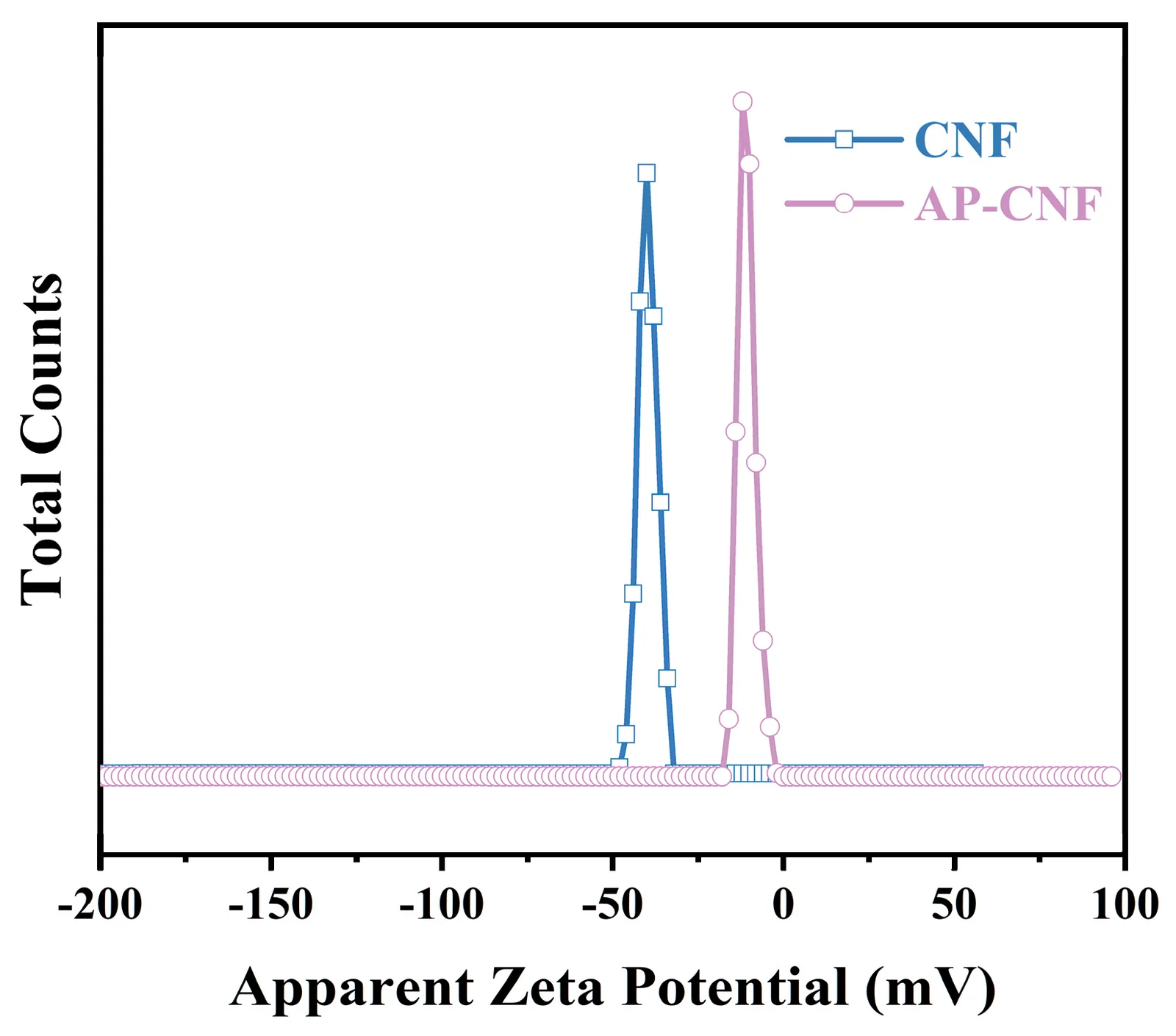
Zeta potential test results of CNF and AP-CNF aerogels.

**Figure 7:**
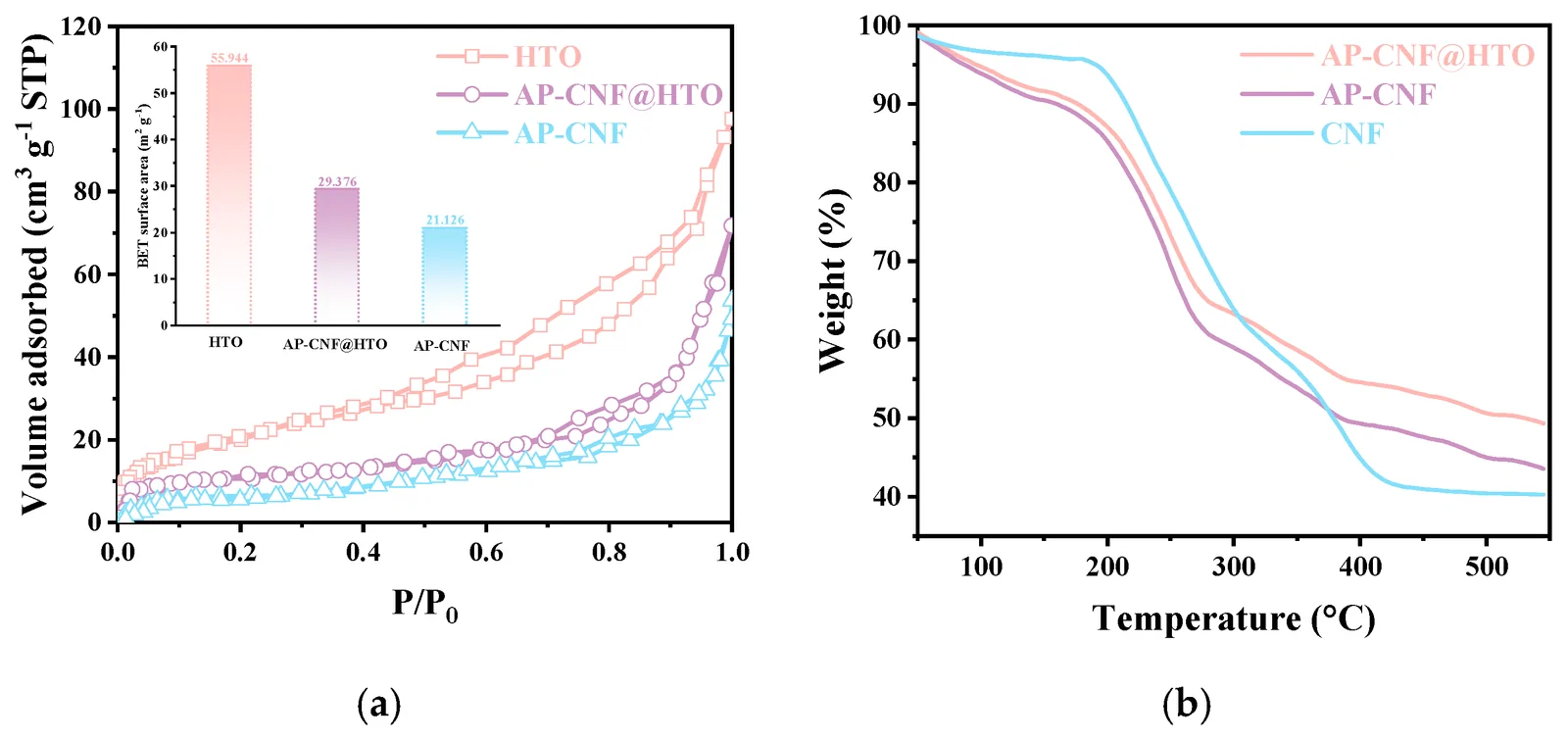
(**a**) BET specific surface area of HTO, AP-CNF, and AP-CNF@HTO. (**b**) TGA analysis of CNF, AP-CNF, and AP-CNF@HTO.

**Figure 8:**
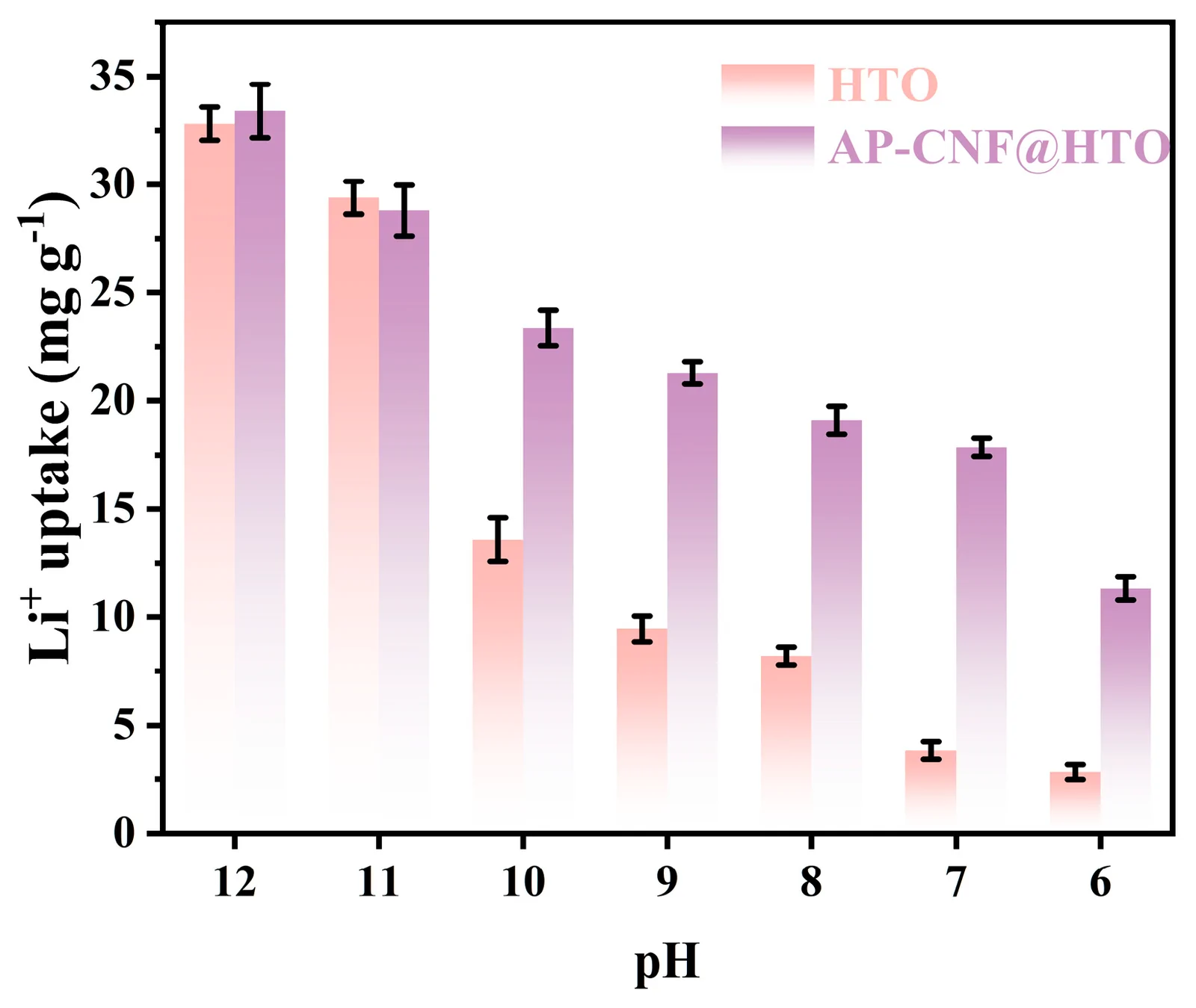
Comparison of the adsorption performance of HTO and AP-CNF@HTO in different acid-base environments.

**Figure 9:**
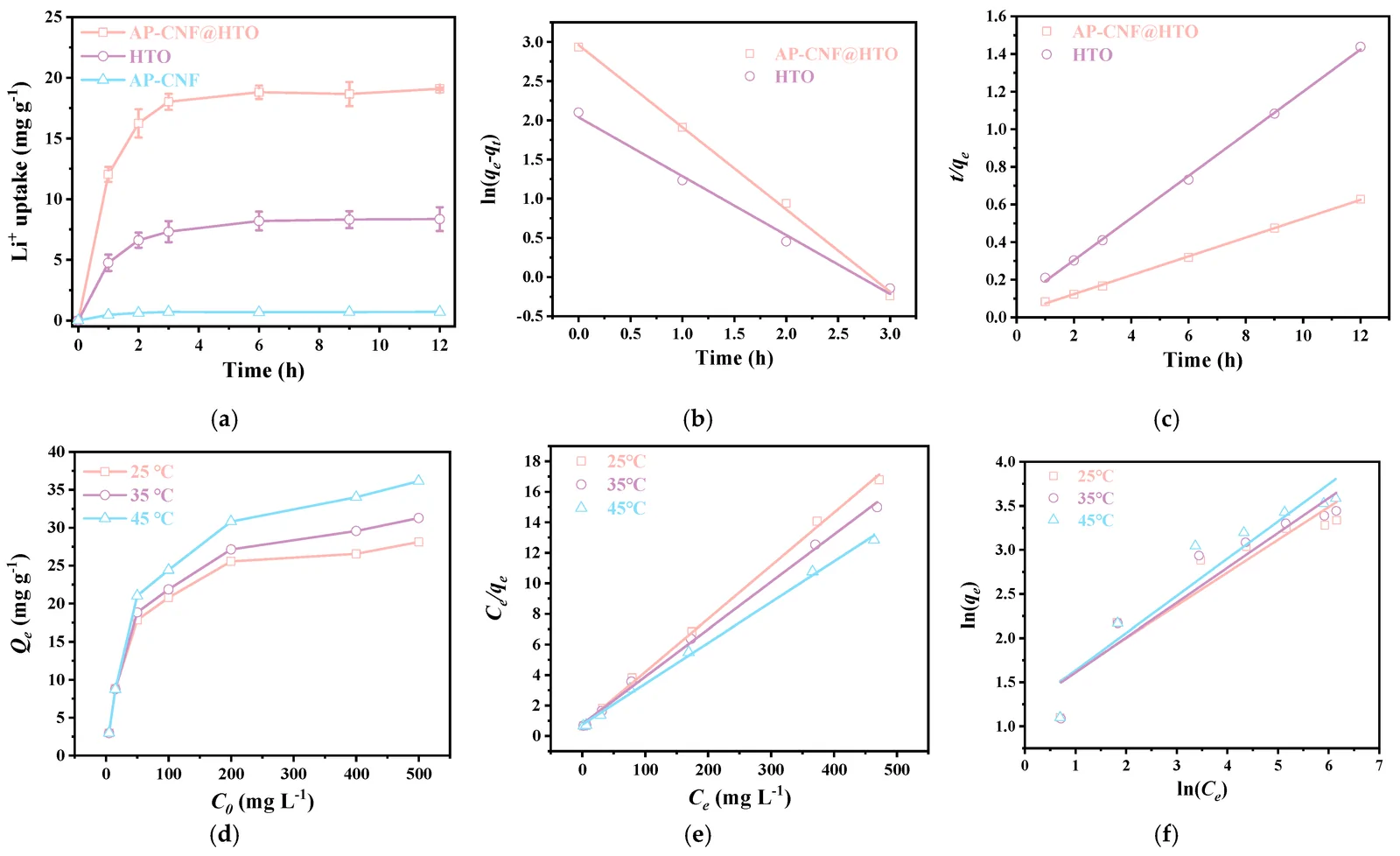
(**a**) The variation of lithium adsorption capacity, (**b**) Linear fitting of pseudo-first-order kinetics, (**c**) Linear fitting of pseudo-second-order kinetics, (**d**) Variation of equilibrium adsorption capacity with initial Li^+^ concentration at different temperatures, (**e**) Linear fitting of Langmuir model, and (**f**) Linear fitting of Freundlich model.

**Figure 10:**
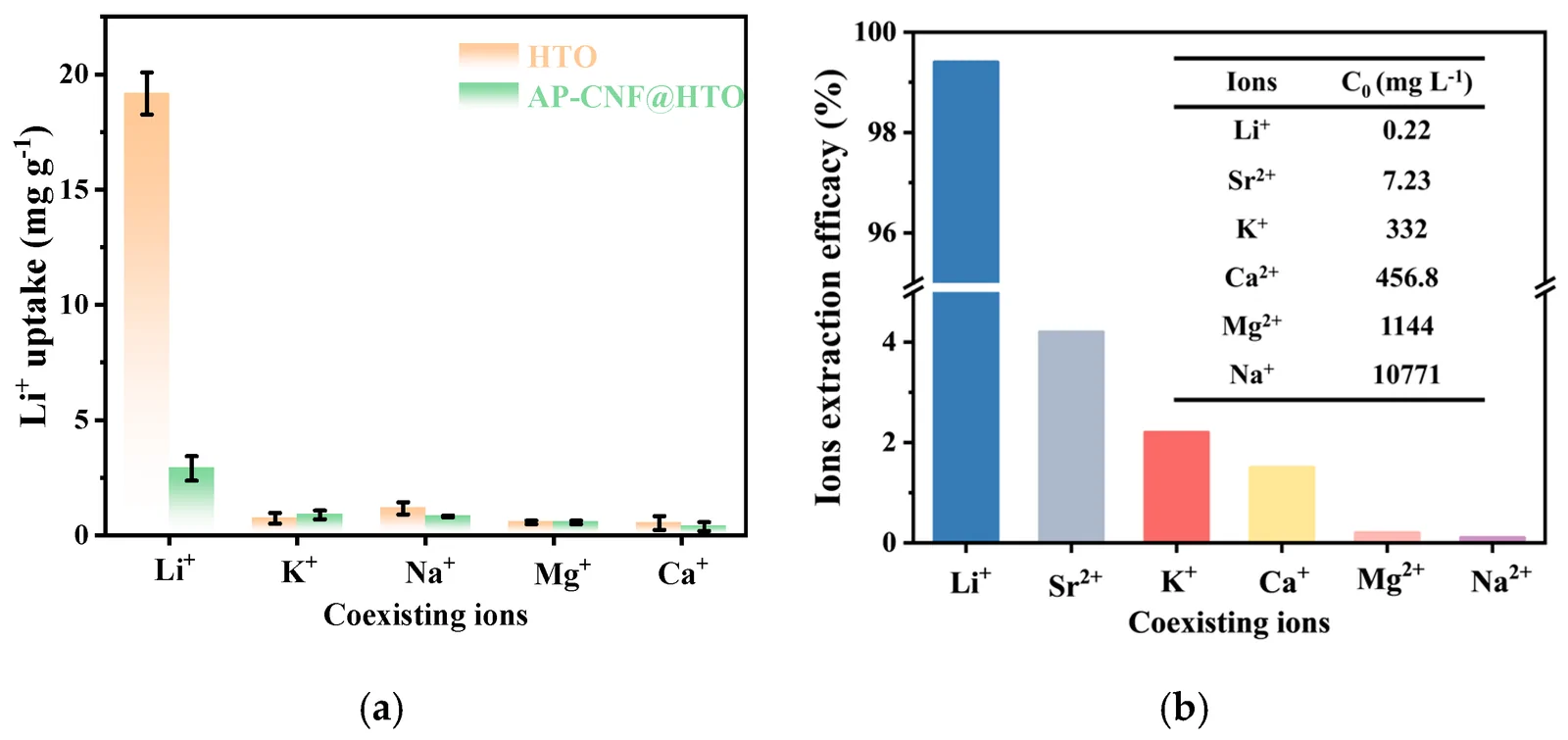
(**a**) Adsorption capacity of AP-CNF@HTO toward different metal ions in a multi-ion mixed solution at pH = 8. (**b**) Extraction efficiency of various cations by AP-CNF@HTO in real natural seawater.

## Data Availability

The data presented in this study are available on request from the corresponding author.

## Supplementary Materials

The following supporting information can be downloaded at https://www.mdpi.com/article/10.3390/gels12100873/s1, Figure S1: AFM test of AP-CNF@HTO; Figure S2: Presents the XPS spectra of AP-CNF@HTO; Figure S3: Li 1s XPS spectrum of AP-CNF@HTO after Li^+^ adsorption; Figure S4: FTIR spectrum of AP-CNF@HTO after Li^+^ adsorption; Figure S5: Fitting results of the Weber–Morris intraparticle diffusion model; Figure S6: Fitting results of the Elovich model; Figure S7: Fitting results of the Redlich-Peterson model; Table S1: Pseudo first order and Pseudo second order; Table S2: Langmuir model and Freundlich model. Equations (S1)–(S6): Equations used for adsorption capacity, Weber–Morris intraparticle diffusion, Elovich kinetic analysis, and removal efficiency.

## Abbreviations

The following abbreviations are used in this manuscript:

LTO	Lithium titanate
CNF	Cellulose nanofibril
AP	Ammonium Phytate
HTO	Hydrogen titanium oxide
