# Supplementary material for: Ambient-Dried Cellulose Nanofibril/H2TiO3 Composite Aerogel for Enhanced Lithium Capture at Seawater-Relevant pH
Source: Gels. 2026 Sep 28;12(10):873. doi: 10.3390/gels12100873 (PMC13704011; doi:10.3390/gels12100873)
Supplement: Supplementary file 1 [file gels-12-00873-s001.zip › gels-4564015-supplementary.pdf]

Supplementary Information

Ambient-Dried Cellulose Nanofibril/H_2TiO_3 Composite Aerogel for Enhanced Lithium Capture at Seawater-Relevant pH

Characterization:

A series of systematic characterization techniques were employed to evaluate the structural, morphological, electrochemical, and adsorption properties of the prepared materials. Fourier transform infrared spectroscopy (FTIR, Nicolet iN10) was utilized to record molecular vibration characteristic peaks and verify the intermolecular crosslinking and functional group variations of different samples. The surface charge properties of the materials before and after modification were measured using a Malvern Zetasizer Pro nanoparticle and zeta potential analyzer. X-ray diffraction (XRD, Shimadzu XRD-6100) was applied to identify the crystalline phase and analyze the crystal structure of the adsorbents. Nitrogen adsorption–desorption isotherms were acquired via a Micromeritics ASAP 2460 specific surface area analyzer to investigate the pore structure and specific surface area characteristics. Thermogravimetric analysis (TGA, Netzsch TG 209 F1 Libra) was performed under a nitrogen atmosphere to assess the thermal stability and thermal decomposition behavior of the aerogel composites. The microscopic morphology and surface microstructure were observed by field emission scanning electron microscopy (SEM, ZEISS Gemini 300) with high resolution. Atomic force microscopy (AFM, Bruker) was used to analyze the surface roughness and three-dimensional morphological features of the materials. Surface elemental composition and chemical states were determined using X-ray photoelectron spectroscopy (XPS, Thermo Scientific K-Alpha). Finally, inductively coupled plasma optical emission spectrometry (ICP-MS, PerkinElmer Avio 550 Max) was adopted to quantitatively detect the concentrations of lithium and other coexisting metal ions in solution during adsorption experiments.

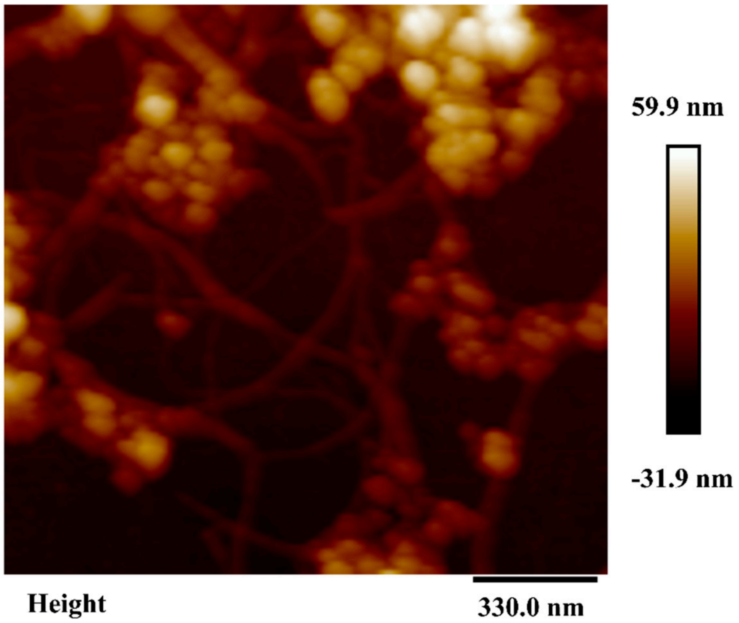

Figure S1. AFM test of AP-CNF@HTO.

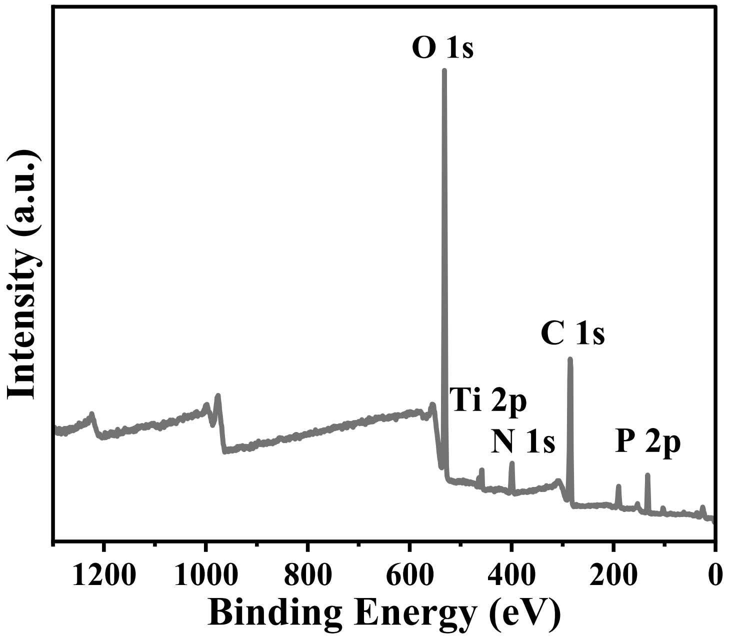

Figure S2. Presents the XPS spectra of AP-CNF@HTO.

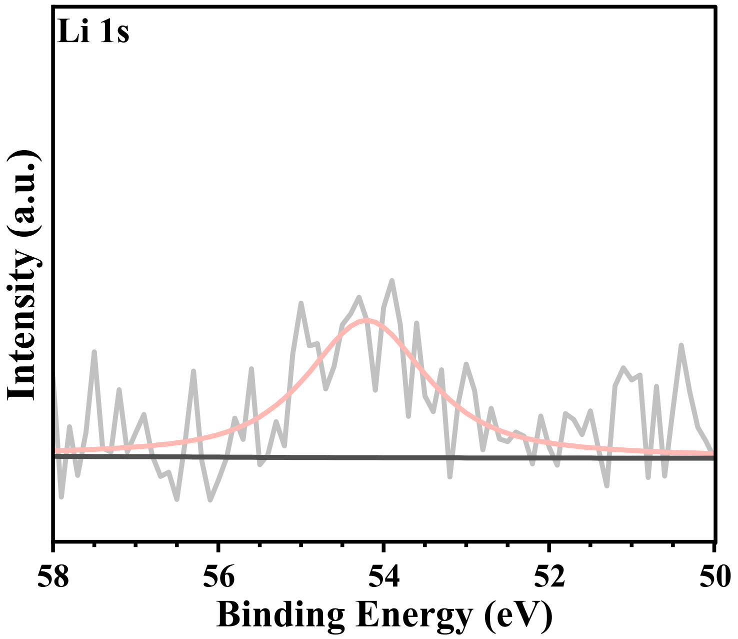

Figure S3. Li 1s XPS spectrum of AP-CNF@HTO after Li^+ adsorption.

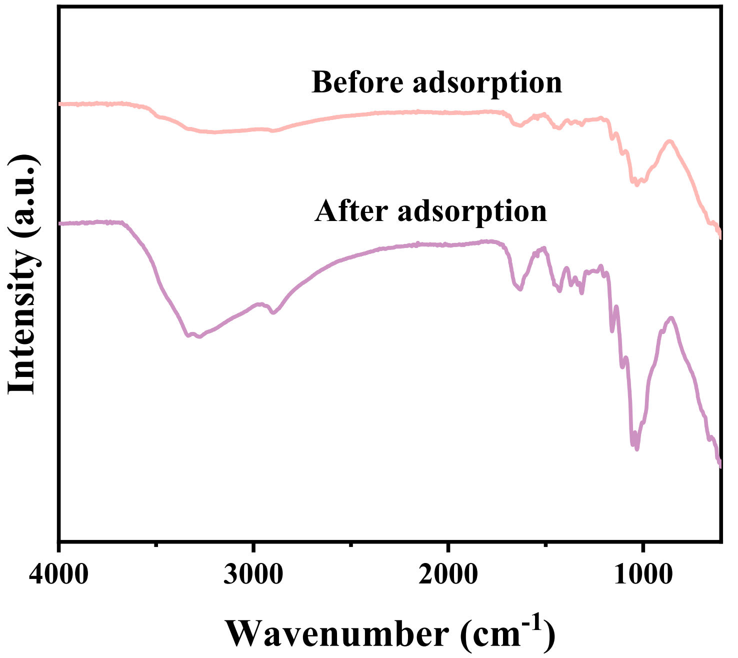

Figure S4. FTIR spectrum of AP-CNF@HTO after Li^+ adsorption.

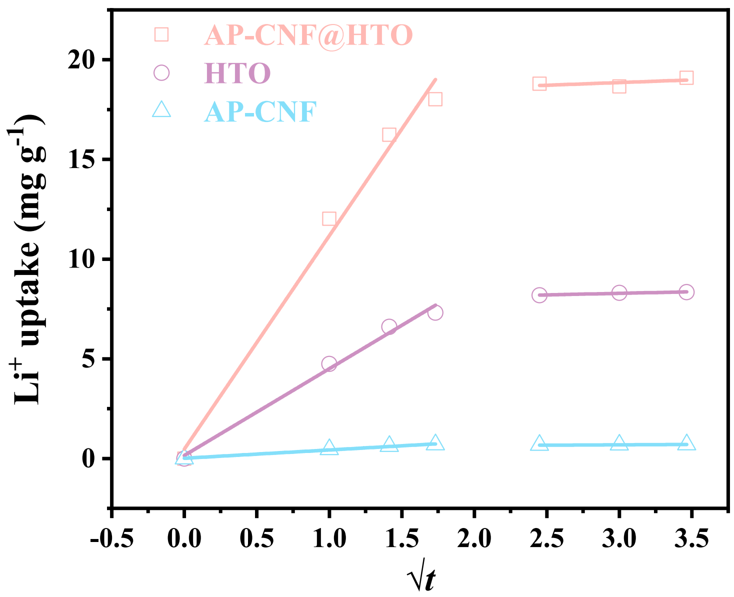

Figure S5. Fitting results of the Weber–Morris intraparticle diffusion model.

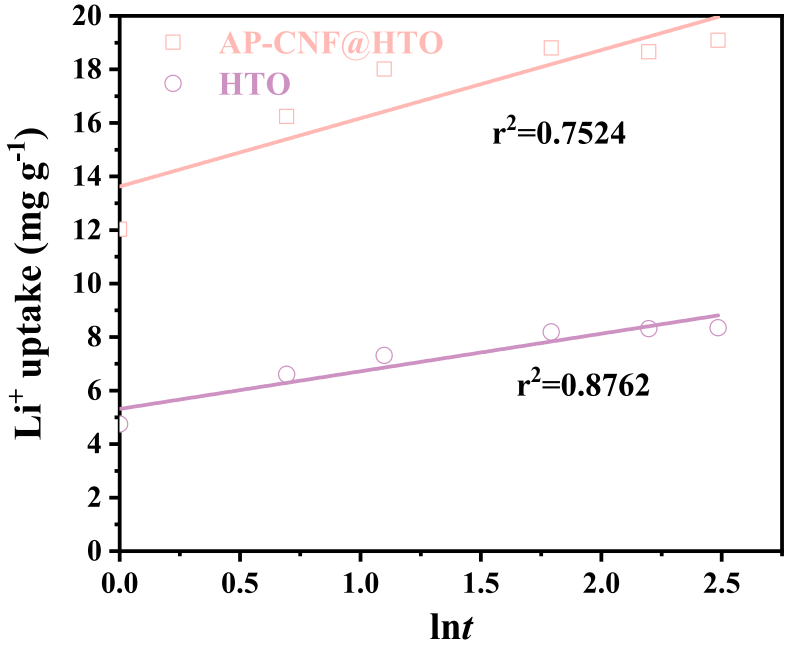

Figure S6. Fitting results of the Elovich model.

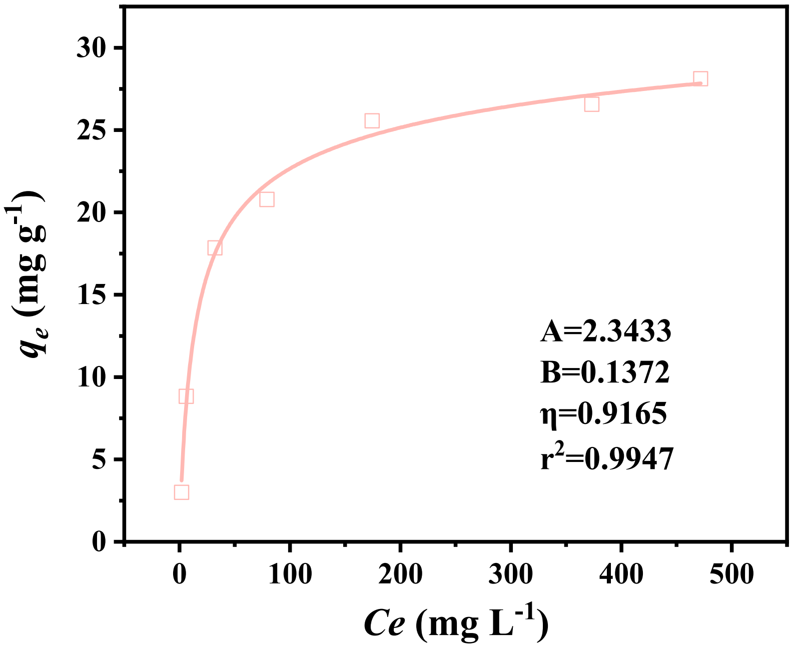

Figure S7. Fitting results of the Redlich-Peterson model

Equations:

$$q_e = \frac{(C_0 - C_e)V}{m} \tag{S1}$$

$$q_t = k\sqrt{t} + C \tag{S2}$$

$$q_t = \frac{1}{\beta} ln\, t + \frac{1}{\beta} ln\, \alpha\beta \tag{S3}$$

$$R(\%) = \frac{C_0 - C_e}{C_0} * 100\% \tag{S4}$$

$$q_e = \frac{AC_e}{1 + BC_e^{\eta}} \tag{S6}$$

α: initial adsorption rate constant

β: constant related to surface coverage

A, B: R-P constant

η: Dimensionless exponent, physical meaning requires 0<η<1

Table S1. Pseudo first order and Pseudo second order.

Material	Pseudo First Order			Pseudo Second Order		
	q_e (mg g^{-1})	$k_1 \times 10^{-3}$ (min^{-1})	r^2	q_e (mg g^{-1})	$k_2 \times 10^{-3}$ (min^{-1})	r^2
AP-CNF@HTO	19.31	17.48	0.9983	19.95	1.77	0.9992
HTO	7.68	12.52	0.9933	8.93	2.06	0.9991

Table S2. Langmuir model and Freundlich model.

Temperature	Langmuir Isotherm			Freundlich Isotherm		
	q_m (mg g^{-1})	$k_1 \times 10^{-3}$	r^2	k_2	n	r^2
25°C	28.75	48.45	0.9983	3.51	2.69	0.8883
35°C	32.23	39.71	0.9977	3.37	2.53	0.9000
45°C	37.43	35.75	0.9974	3.36	2.37	0.9056
